# Uncovering the Role of Distal Regions in PDK1 Allosteric Activation

**DOI:** 10.1021/acsbiomedchemau.5c00025

**Published:** 2025-03-24

**Authors:** Nagaraju Mulpuri, Xin-Qiu Yao, Donald Hamelberg

**Affiliations:** † Department of Chemistry, Georgia State University, Atlanta, Georgia 30302-3965, United States; ‡ Department of Chemistry, University of Nebraska at Omaha, Omaha, Nebraska 68182-0266, United States

**Keywords:** kinases, PDK1, allostery, difference contact network analysis (dCNA), multiensemble contact network analysis, molecular dynamics simulations

## Abstract

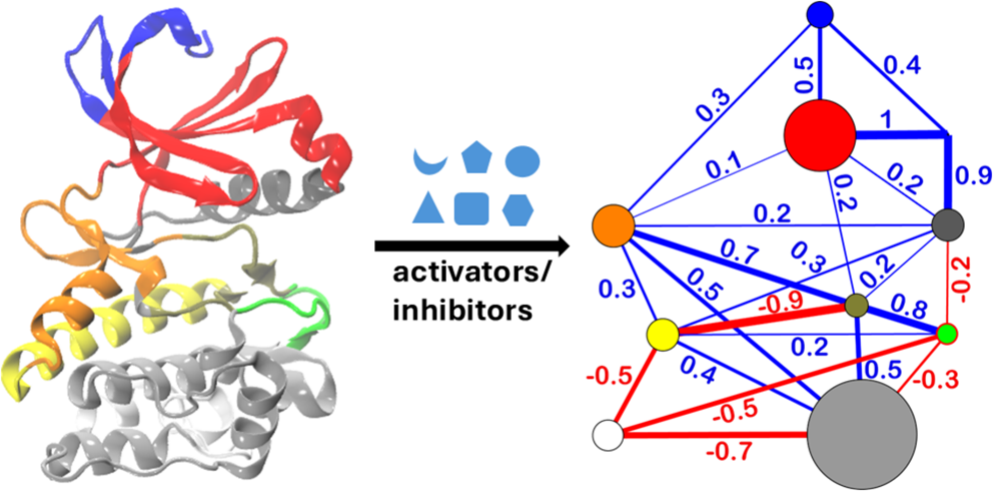

Allosteric regulation is a pivotal mechanism governing a wide array of cellular functions. Essential to this process is a flexible biomolecule allowing distant sites to interact through coordinated or sequential conformational shifts. Phosphoinositide-dependent kinase 1 (PDK1) possesses a conserved allosteric binding site, the PIF-pocket, which regulates the kinase’s ATP binding, catalytic activity, and substrate interactions. We elucidated the allosteric mechanisms of PDK1 by comparing conformational ensembles of the kinase bound with different small-molecule allosteric modulators in the PIF-pocket with that of the modulator-free kinase. Analysis of over 48 μs of simulations consistently shows that the allosteric modulators predominantly influence the conformational dynamics of specific distal regions from the PIF-pocket, driving allosteric activation. Furthermore, a recently developed advanced difference contact network community analysis is employed to elucidate allosteric communications. This approach integrates multiple conformational ensembles into a single community network, offering a valuable tool for future studies aimed at identifying function-related dynamics in proteins.

## Introduction

Allostery is a crucial phenomenon in biology, allowing proteins and other biological macromolecules to be regulated at a distal (allosteric) site from the functional site (e.g., the active site in enzymes). The structural basis of allostery is that proteins can exist in an ensemble of distinct, dynamic conformational states, with relative stabilities of these states closely associated with a specific protein function. Ligand binding, for example, at the allosteric site stabilizes particular states within this ensemble, altering the conformational ensemble and resulting in functional changes.^1−3^ Depending on the nature of the ligand, these effectors can either enhance the protein’s activity (allosteric activators) or inhibit it (allosteric inhibitors). The ability to control protein function through allostery also provides a powerful tool for understanding and manipulating cellular pathways. By selectively modulating the activity of a single protein among homologous proteins, researchers can gain insights into the complex networks that govern cellular behavior.

In drug discovery, there has been a growing interest in targeting allosteric sites to improve the specificity of therapeutic compounds. Allosteric drugs offer several advantages over traditional, orthosteric drugs that target the active site directly. Allosteric sites tend to be less conserved across protein families, offering greater selectivity and minimizing the risk of off-target effects.^4−6^ Even though advances in experimental and computational techniques, such as high-throughput screening,^7−10^ directed evolution,^11^ and computational modeling,^12−14^ have provided new insights for understanding allostery, many aspects of how distant parts of a protein communicate and how conformational changes propagate through the protein remain unclear. Here, we explored the structural network involved in ligand binding to allosteric sites, using PDK1 as a case study.

**Figure 1:**
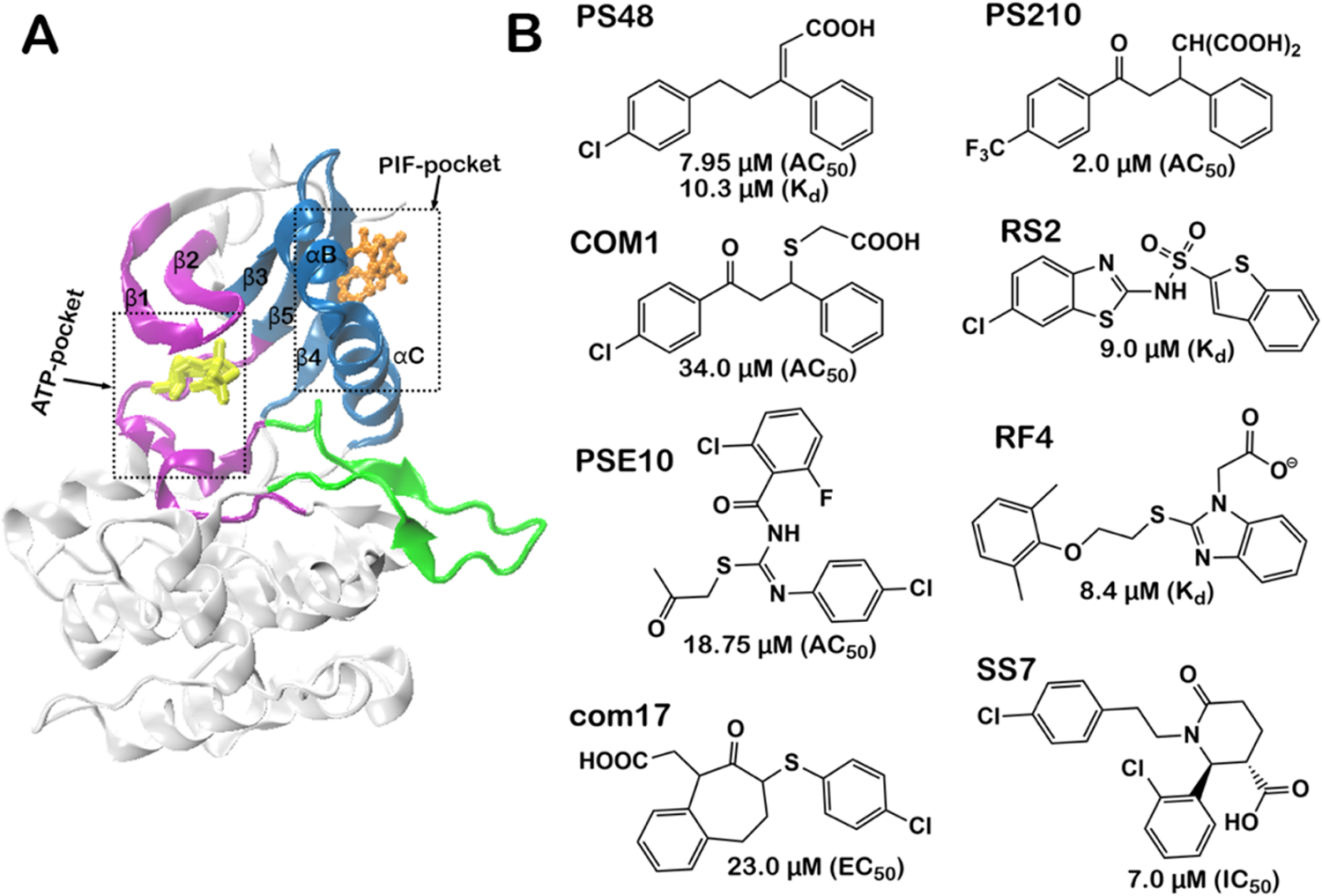
(A) Crystal structure of the catalytic domain of PDK1 in complex with ATP (yellow atoms in stick model; ATP binding site in purple color) binding at the active site and allosteric modulators (orange atoms in ball and stick model, PIF-pocket in blue color) binding at the PIF-pocket allosteric site (PDB: 3HRF). The activation loop is in green color. (B) Chemical structures of allosteric modulators considered in this study (PS48, PS210, COM1, RS2, PSE10, RF4, SS7, com17) with their allosteric activity values (AC_50_, IC_50_, EC_50_, or *K*_d_) in parentheses. The *K*_d_, IC_50_, and EC_50_ instead of the AC_50_, are reported for modulators RS2, RF4, com17, SS7.

Phosphoinositide-dependent kinase-1 (PDK1) is a critical enzyme that plays a central role in several signaling pathways regulating cellular processes such as metabolism, growth, proliferation, and survival.^15−19^ PDK1, belonging to the AGC (protein kinases A, G, and C) kinase family, is integral to the activation of various other AGC kinases, including protein kinase B (PKB/Akt),^17^ p70 ribosomal S6 kinase (S6K),^15^ and serum and glucocorticoid-inducible kinase (SGK).^18,19^ PDK1 is activated via the PI3K pathway, where extracellular signals such as insulin and growth factors trigger the production of phosphatidylinositol 3,4,5-trisphosphate (PIP3), leading to subsequent activation of PDK1 and its downstream targets.^20−23^ PDK1 comprises a catalytic kinase domain and a pleckstrin homology (PH) domain that facilitates its localization to the plasma membrane in response to PIP3 production.^20^ The small lobe of the PDK1 kinase domain contains the PIF-pocket, located at αB-helix, αC-helix, and β-sheet that is separated from ATP binding site (see Figure 1).^24−26^ The structural integrity of PIF-pocket and its interactions with various ligands are fundamental to the allosteric regulation of PDK1. The PIF-pocket naturally interacts with PIFtide, a 24-amino acid polypeptide (REPRILSEEEQEMFRDFDYIADWC) derived from the hydrophobic motif (HM) of PRK2.^24^ At the molecular level, allosteric regulation entails the interaction between distant sites within a biomolecule, resulting in cooperative substrate binding or alterations in catalysis when an effector binds.^27,28^ Disruptions in allosteric regulation in PDK1 are associated with many human diseases, such as cancer, diabetes, cardiovascular diseases, and neurological disorders.^29−32^ Therefore, understanding the mechanisms behind allosteric regulation in PDK1 is crucial for comprehending both physiological and pathological processes, providing valuable insights for drug discovery. Additionally, the significance of allosteric effects is gaining substantial recognition in the modern rational protein design.^33^

Recent research has focused on the design and synthesis of small-molecule allosteric modulators targeting the PIF-pocket of PDK1.^34−45^ These compounds can function as either activators or inhibitors, depending on their specific interactions with the PIF-pocket. While some modulators enhance PDK1 activity by inducing beneficial conformational changes, others inhibit its function by preventing activation-associated structural rearrangements. Crystallographic studies have provided detailed insights into the binding mechanisms of these allosteric modulators.^35,36,39,40^ For example, the binding of small compounds to the PIF-pocket results in the stabilization of key structural elements of PDK1, including helix αC and the DFG motif, which are critical for its activation.^36^ Additionally, recent studies highlight the critical role of the PDK1 linker region in modulating its activity through interaction with the PIF-pocket. The linker stabilizes key interactions, such as the Glu130-Lys111 salt bridge, facilitating the transition from an autoinhibited to an active state.^46^ Furthermore, regulatory motifs within the linker enhance trans-autophosphorylation and activation, fine-tuning PDK1 function.^47^ These studies revealed that allosteric modulators induce conformational changes not only at the PIF-pocket but also at distal sites, such as the ATP binding site and the activation loop, highlighting the interconnected nature of PDK1′s regulatory mechanisms.

A wide range of computational techniques has been developed to uncover the atomic-level mechanisms behind allosteric regulation. Several of these techniques utilize network modeling and graph theory to reveal the connections between protein structure and function.^48−54^ Recently, we have proposed difference contact network analysis (dCNA) and applied the method to distinct biological systems.^55−57^ Unlike traditional network methods, dCNA compares distinct conformational ensembles representing different functional states, making it more sensitive in detecting function-related conformational dynamics. Using this method, we have decoded the allosteric communication pathway in Cyclophilin A^58^ and elucidated the allosteric effect elicited by the isomerization in Cyclophilin D upon binding to p53 DNA binding domain.^59^ We have also further enhanced dCNA to allow comparing more than two ensembles.^60^ In this updated method, named multiensemble contact network analysis, functional dynamics are identified by examining the correlation between local conformational changes and functional outcomes measured by experimental data, such as inhibition constants. We have applied this method to investigate the impact of HIV-1 protease mutations on drug resistance.^60^

In this study, we analyze the dynamics of PDK1 kinase, both in its modulator-free form (without allosteric modulators) and in complex with various allosteric modulators, using multiple microsecond-long MD simulations (Figure 1). We employ residue–residue contact analysis to compare the dynamical changes observed in the modulator-bound complexes with those in the modulator-free enzyme. This approach provides detailed insights into the atomistic mechanisms underlying the allosteric activity of the modulators. Crucial allosteric residues of PDK1 are identified and evaluated in relation to previously published findings.^35,37,40,45,61,62^ Overall, the study provides insights at the atomic and residue levels into how allosteric regulations by various modulators and chemical modifications (mutations) of the enzyme can either facilitate or impede catalysis.

## Computational Methods

### System Preparation

A 1.90 Å resolution crystal structure with PDB ID 3HRF(35) was used to carry out the simulations of the allosteric modulator-bound PDK1. Crystallographic water molecules and ATP were retained, while most allosteric modulators were modeled in the PIF-pocket using other crystallographic structures containing specific modulators. Specifically, the allosteric modulators RS2, RF4, SS7, PSE10 were derived from corresponding PDB IDs 4RQV,^37^4XX9,^39^5ACK,^38^ and 5LVO,^40^ respectively, whereas allosteric modulators COM1^34^ and PS210^36^ were derived from PS48.^35^ All these structures were superimposed onto 3HRF before the coordinates of the modulators were extracted. For the modulator com17, there is no available crystallographic structure. Therefore, AutoDock Vina^63,64^ was used to dock com17^44^ into the PIF-pocket of PDK1. Gauss View 3.09^65^ was used to build the modulators shown in Figure 1. The modulators represent a collection of nonpeptidomimetic PDK1 allosteric activators from the literature, with each activator representing the compound with the highest activity within its class.

### Docking Procedure

The nonpolar hydrogen atoms were combined with their corresponding heavy atoms. Gasteiger charges were applied to both the protein and allosteric modulator using AutoDockTools (ADT). The allosteric modulator, com17 (Figure 1) was docked into the 3HRF protein structure. The docking parameters were specified as follows: the grid spacing was 1.0 Å, the box size was 25 Å in each dimension, and the box was centered to encompass all active site residues. The maximum number of binding modes was set to 10. The allosteric modulator configuration with the lowest binding energy in the active site was identified as the optimal binding mode for that conformation of PDK1. This most likely binding mode was then used for molecular dynamics simulation of the PDK1-allosteric modulator complex.

### Molecular Dynamics Simulations

The molecular dynamics (MD) simulations utilized the AMBER 16 suite of programs^66^ in conjunction with the AMBER ff14SB force field parameters,^67^ which are a modified version of the Cornell et al. force field.^68^ To enhance accuracy, we reoptimized the ω-torsion angle parameters as previously demonstrated.^69^ The systems were solvated in a periodic octahedron box filled with pre-equilibrated TIP3P water molecules.^70,71^ To ensure an appropriate separation between the box faces and the solute, a minimum distance of 10 Å was set. The original water molecules from the crystallographic structure were retained. To maintain system neutrality, Na^+^ or Cl^–^ ions were added as needed to neutralize the overall net charge.^72^ Energy minimization was conducted through 5000 steps, consisting of 3000 steps using the steepest descent method followed by 2000 steps using the conjugate gradient method. Harmonic restraints were applied to the solute atoms during minimization, with the force constant for positional restraint gradually decreasing from 500 to 0 kcal·mol^–1^·Å^–2^ over five rounds of minimization. The systems were heated from 100 to 300 K using a Langevin thermostat with a collision frequency (γ) of 1.0 ps^–1^, time scale 500 ps under NVT conditions with 1 fs time step, along with positional restraints on the solute. The force constant for the restraints was adjusted in five rounds from 500, 300, 100, 50, to 5 kcal·mol^–1^·Å^–2^ respectively. Equilibration was carried out for 1 ns for each system, utilizing a 2 fs time step and no restraint under NPT conditions (300 K, 1 bar). The Monte Carlo barostat with a coupling constant (τp) of 1.0 ps was used. Long-range electrostatic interactions were computed using the particle-mesh Ewald (PME) summation method,^73^ while short-range nonbonded interactions were treated with a cutoff of 9 Å. Bonds involving all hydrogen atoms were constrained using the SHAKE algorithm^74^ and the simulation snapshots were saved at intervals of 1 ps. Each production simulation was extended to 2.0 μs. Each simulation was replicated 3 times, these accumulated a total of 48.0 μs simulation data. The last 1.5 μs of each trajectory were analyzed and averaged to evaluate various conformational characteristics of PDK1. Root-mean-square fluctuations (RMSF), Root-mean-square deviations (RMSD), and residue–residue contact statistics were used for comparative analysis of the MD trajectories. RMSF calculations were performed using CPPTRAJ of AmberTools,^75^ focusing on the backbone atoms (N, Cα, C, and O). Prior to RMSF calculations, all conformations were superimposed onto the first frame of the simulation based on backbone atoms. The RMSF values were then averaged for each residue over the entire simulation period.

### Contact Network Analysis

Contact statistics were computed following the methodology outlined in Doshi et al.^76^ A contact is defined when any two heavy atoms from different residues (i to i + *n*, where *n* ≥ 3) are within 4.5 Å of each other. The probability of contact formation (*p*_c_) during simulations was determined, and contacts that formed less than 10% of the time across trajectories were excluded, as their occurrence was deemed too infrequent for significant comparison between WT and mutant variants (*p*_c_ close to 0).

Difference contact network analysis (dCNA) was conducted as previously described.^56^ First, we used contacts with a high probability of formation (*p*_c_ ≥ 0.9) across all trajectories to build a consensus contact network. The Girvan–Newman’s algorithm^77^ and network modularity analysis^78^ were then employed to identify communities based on the consensus network. Net contact probability changes between communities from one system to the other were computed as the final output.

Multiensemble contact network analysis was conducted using available experimental functional data, following the procedure described previously.^60^ The analysis focused on both residue and community levels, utilizing communities identified in dCNA. We calculated Pearson’s correlation coefficient between individual residue–residue contact probabilities or net intercommunity contact probabilities and the logarithm of the allosteric activation constant (*K*) values of the allosteric modulators. Key regions potentially impacting protein function upon perturbation (e.g., mutations and ligand binding) were predicted to be those exhibiting high absolute correlation values. The allosteric activation constant, *K*, for each modulator against modulator free was measured in prior experimental studies: PS48 (AC_50_ = 7.95 μM, *K*_d_ = 10.3 μM),^35^ PS210 (AC_50_ = 2.0 μM),^36^ COM1 (AC_50_ = 34.0 μM),^34^ RS2 (*K*_d_ = 9.0 μM),^37^ PSE10 (AC_50_ = 18.75 μM),^40^ RF4 (*K*_d_ = 8.4 μM),^39^ com17 (EC_50_ = 23.0 μM),^44^ SS7 (IC_50_ = 7.0 μM).^38^ Bio3D^79−81^ and igraph^82^ R packages were used to generate the residue communities and correlation analysis figures. Residue-wise correlations between the communities were calculated using contact statistics.

## Results and Discussion

Extensive multimicrosecond MD simulations were used to characterize the internal dynamics of PDK1 when allosteric modulators were bound to PIF-pocket. These contain three independent 2-μs simulations for each modulator and a total of 48 μs simulations for 8 modulators (Figure 1). Residue-wise averaged RMSF of the PDK1 complex with modulators depicts significant fluctuation in many regions of the structure (Figure S1). However, there is no correlation between the magnitude of fluctuation and the allosteric activity of the modulators: At each peak of RMSF fluctuation, the order of allosteric activity of the modulators does not follow the order of the RMSF values. Contact network analysis sheds light on conformational changes that are more consistent with the allosteric activity of the modulators. The dynamic properties were calculated using average contact statistics. We used multiensemble contact network analysis and difference contact network analysis methods to investigate dynamically coordinated regions in all simulations. Finally, the potential regions predicted to allosterically modulate PDK1 function through our contact network analysis were validated by comparing them with available experimental mutational studies. Our work also elucidates the allosteric mechanisms underlying these mutations.

### Difference Contact Network Analysis Provides Insights into Mechanisms Underlying Modulator Specific Allosteric Effects

To gain a clearer insight into the conformational changes reflected in residue–residue interactions, we have recently developed a method, called dCNA, for comparative protein structure network analysis and applied it to various biological system like cyclophilins, Pin1, NF-κB/DNA complex, acid-β-glucosidase, and d-arginine dehydrogenase.^55−60^ In dCNA, conformational changes are analyzed by measuring the variations in the formation and disruption of residue–residue contacts. This method was employed to compare the conformational ensembles of modulator-bound complexes (PDK1 + ATP + modulators) with those of the modulator-free (PDK1 + ATP). Consequently, the observed contact formations and disruptions are statistically significant, described by their probability of occurrence. The underlying hypothesis is that the allosteric signals observed in modulator-bound complexes are indicative of both extensive backbone or domain movements and minor side-chain adjustments. Difference contact network analysis reveals a residue community structure that is common across PDK1 when different modulators bind to the allosteric site, PIF-pocket. Eight modulator-bound complexes are compared with the modulator-free system. These eight systems represent different magnitudes of allosteric regulations in PDK1 upon binding to the PIF-pocket. PS210 has the highest allosteric activity, whereas COM1 has the lowest activity (Figure 1).

**Figure 2:**
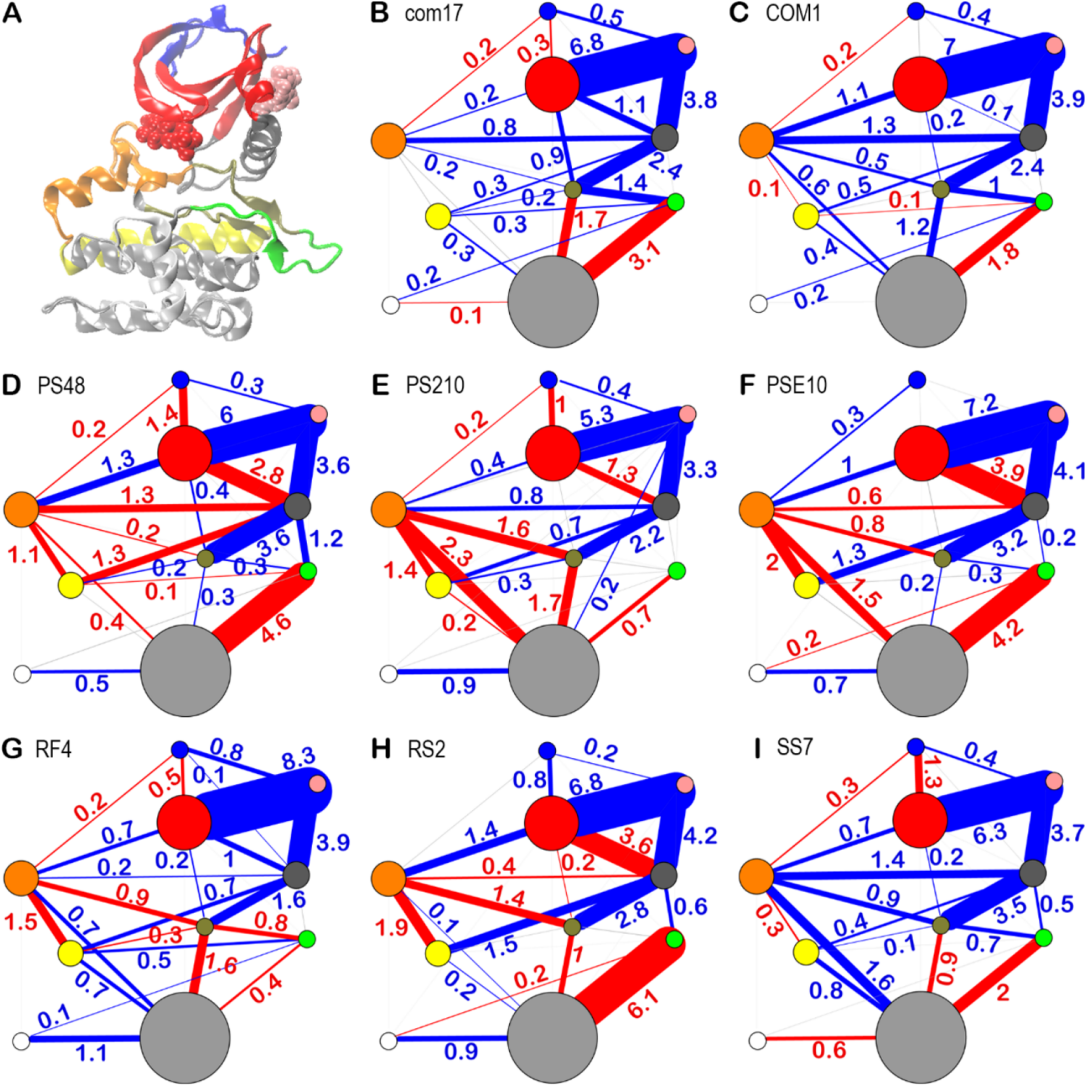
Difference contact network analysis reveals modulator specific dynamical changes between segments of residues. (A) Residues are grouped based on a community analysis of the consensus contact network across all modulators. Residues in the same group are mapped in the PDK1 structure using the same color. (B–I) Community networks are represented by colored circles (as in panel A) and lines. The radius of circles is proportional to the number of residues within the corresponding community. Lines connecting the circles depict changes in overall contact probabilities (Δ*f*) between the communities from unbound modulator to bound modulator. Blue and red lines indicate significantly (|Δ*f*| ≥ 0.1) enhanced and reduced contact probabilities, respectively, upon modulator binding. Lines with |Δ*f*| < 0.1 are shown in gray. Line width corresponds to |Δ*f*|, with values provided for lines where |Δ*f*| ≥ 0.1.

The consensus network identified ten distinct residue communities or subdomains (Figure 2). Intriguingly, the modulator binding PIF-pocket is partitioned into two communities (Figure 2A, gray and red), where the modulator itself is considered as a different community (Figure 2A, pink). ATP binding pocket is partitioned into three communities (Figure 2A, red, orange and tan) and ATP belongs to the red community. The activation loop is partitioned into two communities, green and tan (Figure 2A), which account for the major flexibility of PDK1. The results reveal that substantial contact changes (with an absolute probability difference of contact formation ≥0.1) occur throughout the PDK1 structure when comparing modulator-bound and modulator-free forms (Figure 2). Notably, these significant alterations are predominantly driven by specific helices, such as αB-helix and αC-helix, located on the protein surface near the modulator binding site. These helices undergo structural rearrangements upon modulator binding, contributing to the reorganization of residue communities and contact probability changes within PDK1.

For ten communities, the collective contact changes between modulator-bound and modulator-free forms are summarized and illustrated in a two-dimensional diagram as shown in Figure 2B–I. The community-level difference contact network reveals significant conformational changes involving contact formation and breakage, as indicated by the thick blue and red lines in Figure 2B–I. The most significant change, with a total contact probability difference in the range of 5.3 to 8.3, occurs near the modulator binding site (represented by the red and pink communities in Figure 2B–I), suggesting tightened interactions in the region. The dynamical changes between communities reveal the mechanisms of driving ligand-specific allosteric effects. Four intercommunity regions were analyzed: the areas between the orange and tan (24.5 Å), orange and silver(32.5 Å), orange and yellow(29.3 Å), and tan and silver(32.7 Å), which are far from the modulator community (pink colored community, Figure 2B–I). These regions exhibit the most significant overall changes in community-community contacts. These are either directly connected to or separated by just one community from the red or gray communities, which form strong interactions with the modulator. This is consistent with the experimentally determined allosteric effect of these modulators, where PS210 shows the largest efficacy. There is a key difference between allosteric activators (PS48, PS210, PSE10) and inhibitors (RS2, SS7, RF4). In the case of activators, interactions between the orange and silver communities are disrupted, while in inhibitors, these interactions are formed. Additionally, no change is observed in the interactions between the yellow and silver communities for activators, but such interactions are formed in inhibitors.

### Multi-Ensemble Contact Network Analysis Identifies Key Conformational Changes throughout the Protein Correlated with Modulators Activity

Our previous implementation of the multiensemble contact network analysis on HIV-1 protease^60^ showed that this method offered valuable insights into function-related local conformational changes driven by mutations. Here, we used the same method to understand the functional motions of PDK1 upon binding various allosteric modulators. We performed two sets of analysis: (i) considering all modulators and (ii) excluding the modulators that have only *K*_d_ experimental values. Investigating set (ii) is because *K*_d_ differs from other measurements of the allosteric activity of modulators, such as AC_50_, EC_50_, and IC_50_, and excluding *K*_d_ helps make the functional values more comparable.

#### Multiensemble Contact Network Analysis Using All Modulators

**Figure 3:**
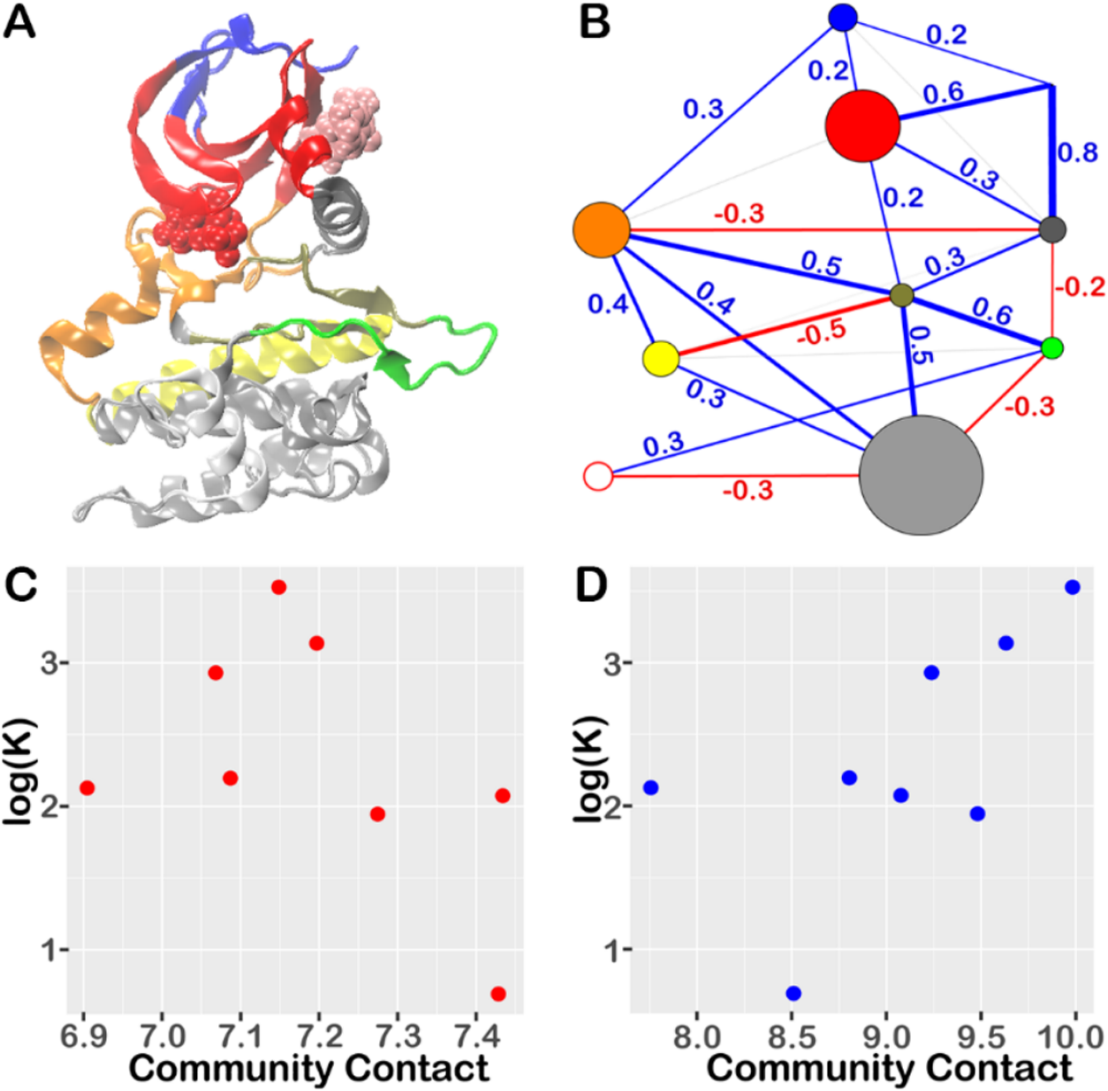
Residue communities and correlation analysis between community-community contacts and the modulator activity profile with all modulators considered here. (A) Mapping of 10 consensus communities, each represented by a distinct color, onto the structure of PDK1 kinase. (B) Vertices represent the communities color-coded as in panel (A). The radius of each vertex corresponds to the number of residues within the community. Lines represent the correlation between the sum of residue–residue contact probabilities between two communities and the logarithm of the allosteric activity (K) values for 8 modulators. Line width reflects the magnitude of Pearson correlation coefficient, with negative and positive correlations indicated by red and blue lines, respectively. Correlations with magnitudes less than 0.1 are shown in gray. (C) Example of detailed view for the negative correlation associated with the line between the yellow and tan communities in panel (B). (D) Example of detailed view for the positive correlation associated with the line between the tan and green communities.

Ten consensus communities identified by dCNA are mapped on the PDK1 kinase structure, as shown in Figure 3A. The number of communities is determined by maximizing the modularity, as depicted in Figure S2. The ATP binding site spans the red and orange communities, whereas the PIF-pocket (allosteric modulator binding site) belongs to the red and gray communities. The red community shared between the two binding sites mostly represents the catalytic residues in the binding pockets. The community network (Figure 3B) indicates that contact changes not only surrounding the active site but at distal sites exhibit significant correlations with the enzyme activity. For example, the net contact probability between the yellow and tan communities, both of which are separated from the active site, exhibits a negative correlation (ρ = −0.5), suggesting that interactions between the two communities get weaker when the enzyme becomes more active. Similarly, the tan and green communities distal from the active site display a positive correlation (ρ = 0.6), indicating that more residue–residue contacts are formed between the two communities when the enzyme activity increases.

The pink community, which solely represents allosteric modulator (Figure 3A), forms significant positive correlations with other communities, especially the red and gray communities that contain the key regulatory elements, αB-helix and αC-helix, showing correlation values 0.6 and 0.8, respectively. This indicates that overall, modulator binding positively modulates enzyme function, and more potent binders cause larger increases in enzyme activity.

The Pearson correlation coefficient at the community level offers a convenient way to identify key function-impacting regions. However, this method may miss regions that potentially affect function but exhibit low correlations due to “outliers”. To recatch these regions, we examine the detailed view of correlation for each pair of communities, which offers insights into the trend of changes behind the correlation coefficient. For community pairs exhibiting significant correlations, the detailed views consistently show concerted changes between community contact and log(K) (Figures 3C,D and S3). However, for orange-red community, even though its correlation coefficient is low (<0.1), their detailed view (Figure S4) reveals an overall negative correlation except for outliers.

The silver community demonstrates diverse interactions with other network communities, which correlate with the modulator’s allosteric activity profile. This suggests that perturbation (e.g., mutations) in this community may influence modulator binding through an allosteric effect. The orange, tan, yellow, and green communities establish contacts with the silver community, with these interactions being either positively or negatively correlated with the logarithm of the modulator’s activity.

#### Multiensemble Contact Network Analysis Excluding Modulators Having Only *K*_d_ Values

**Figure 4:**
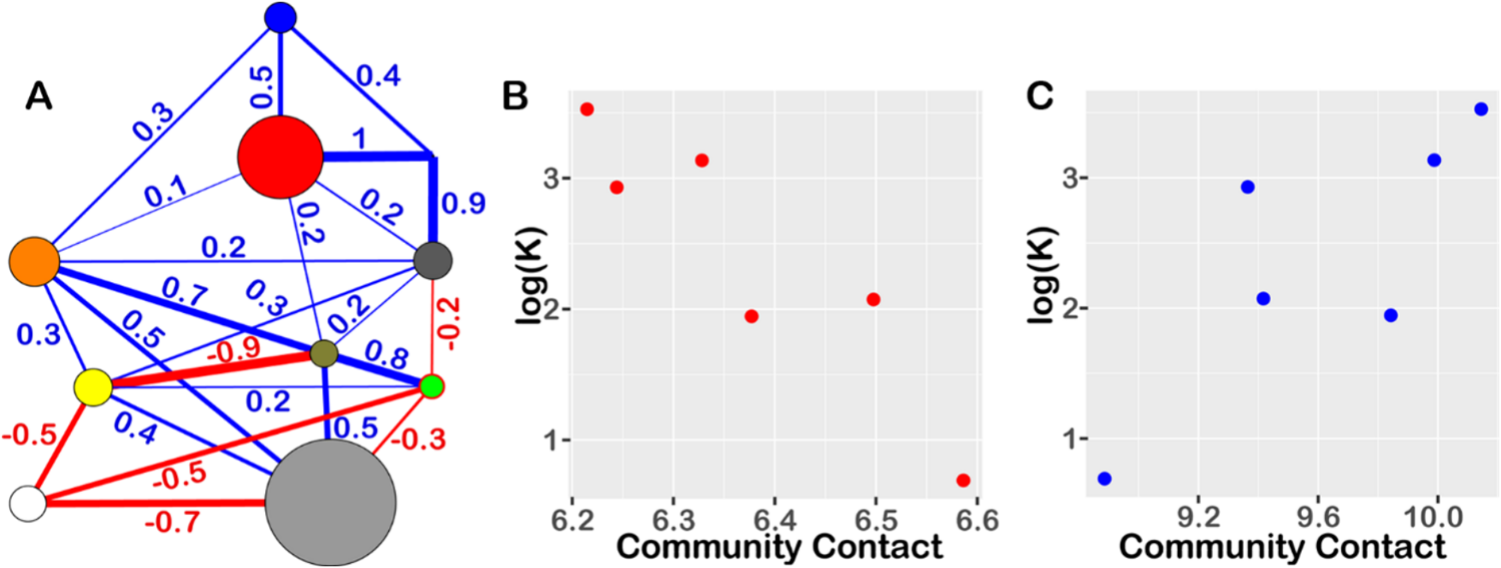
Residue communities and correlation analysis between community-community contacts and the modulators activity profile with six modulators considered here (excluding modulators containing *K*_d_ only). (A) Vertices represent the communities color-coded as in Figure 2 panel (A). The radius of each vertex corresponds to the number of residues within the community. Lines represent the correlation between the sum of residue–residue contact probabilities between two communities and the logarithm of the allosteric activity (*K*) values for the six modulators. Line width reflects the magnitude of Pearson correlation coefficient, with negative and positive correlations indicated by red and blue lines, respectively. Correlations with magnitudes less than 0.1 are shown in gray. (B) Example of detailed view for the negative correlation associated with the line between the yellow and tan communities in panel (A). (C) Example of detailed view for the positive correlation associated with the line between the tan and green communities.

Figure 4 illustrates the community network constructed after excluding modulators with only *K*_d_ values. For comparison, the community partition remains the same as when using all modulators, while the edges between communities are updated using the subset of data. As described above, ten consensus communities were identified by maximizing the modularity (see Figures 3A and S2). The ATP binding site is depicted in red and tan communities, while the allosteric binding site (PIF-pocket) is shown in red and gray communities. The red community, which is shared by both binding sites, consists of catalytic residues within the active-site binding pocket. Figure 4B illustrates a strong negative correlation (ρ = −0.9) indicates a decrease in residue–residue interactions between the yellow and tan communities as the enzyme exhibits increased resistance to allosteric activity. Conversely, Figure 4C shows that increased residue–residue interactions between the tan and green communities are associated with enhanced allosteric activity (ρ = 0.8). The pink community, representing modulators (Figure 3A), displays a strong positive correlation with the red and gray communities containing the key regulatory elements, αB-helix and αC-helix, with correlation values of 1.0 and 0.9, respectively. Contacts between the orange and tan communities and those between the tan and green communities show significant positive correlations with allosteric activity, evidenced by a correlation value of 0.7 and 0.8, respectively (Figure S5). In contrast, contacts between the silver and white communities, as well as those between the yellow and tan communities, exhibit negative correlations with allosteric activity, evidenced by a correlation value of −0.9 and −0.8, respectively. The silver community exhibits diverse interactions with other network communities, correlating with the modulator’s allosteric activity profile. The interactions of the orange, tan, yellow, white, and green communities with the silver community are either positively or negatively correlated with the logarithm of modulator’s activity. Notably, for most community network edges, the correlation trends remain consistent between the networks before and after removing modulators with only *K*_d_, but the correlation strength is enhanced without those modulators.

**Figure 5:**
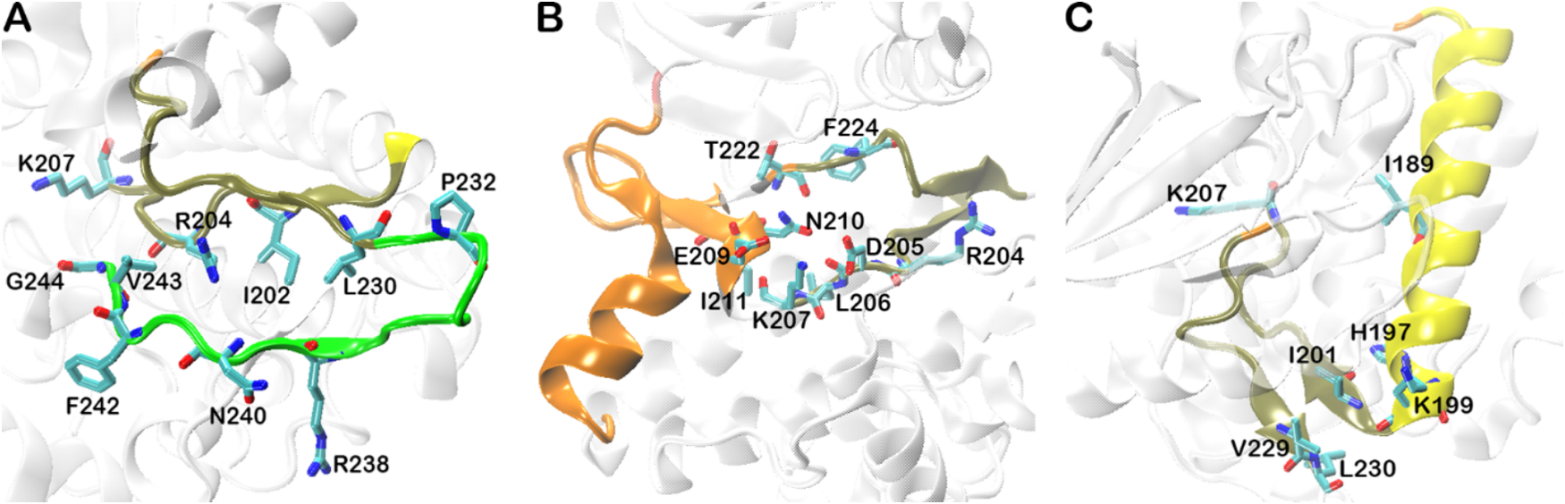
Key residues involved in forming residue–residue contacts exhibiting the top-3 (positive and negative) correlations with modulator activity, calculated using the data that exclude the modulators containing *K*_d_ values only. Residues in the contacts with correlation values >0.5 or <−0.5 are shown here. A full list of residue pairs showing significant correlations for corresponding communities is given in Supporting Information Table S1. (A) Tan-green, (B) orange-tan, (C) tan-yellow communities. Colors of communities are the same as in Figures 2A and 3A.

Figure 5 shows key residues forming residue–residue contacts between communities that exhibit the top 3 positive and negative correlations with the modulator’s activities. A full list of residue pairs showing correlation values greater than 0.5 or less than −0.5 is given for corresponding communities in Supporting Information (Table S1). Residue pairs R204-G244, K207–F242, and R204-N240 from the tan and green communities (Figure 5A) are considered predominant residues for allosteric regulations, as they show correlation values of 0.8, −1.0, and −0.9, respectively. In orange and tan communities, residue pairs K207–I211 and R204-N210 (Figure 5B) have correlation values of 0.7 and −0.6, respectively, which may show a significant effect in allosteric regulations when modulators bind to PIF-pocket. Figure 5C represents tan and yellow communities, in which residue pairs K199–I201 and H197-L230 have correlation values of 0.8 and −0.9, respectively. The tan community is in close proximity to the allosteric site, PIF-pocket, while green and yellow communities are far apart from the PIF-pocket. This indicates that changes occurring in the green and yellow communities influence modulator binding predominantly through allosteric effects. Even though residue-wise correlations are generally weak (with absolute values less than 0.5; data not shown) for white and silver communities, they have a correlation value of −0.7 at the community level, indicating a collective behavior between these two communities in exerting an allosteric effect. Hindie et al. reported that mutations Y288G and Q292A (in the white community), which is at the bottom of the large lobe more than 30 Å away from the PIF-pocket, disrupt the crystallographic packing of the PIF-pocket.^35^ These results support the importance of the white community in allosteric regulation.

### Impact of Mutations on Allosteric Activation

**Figure 6:**
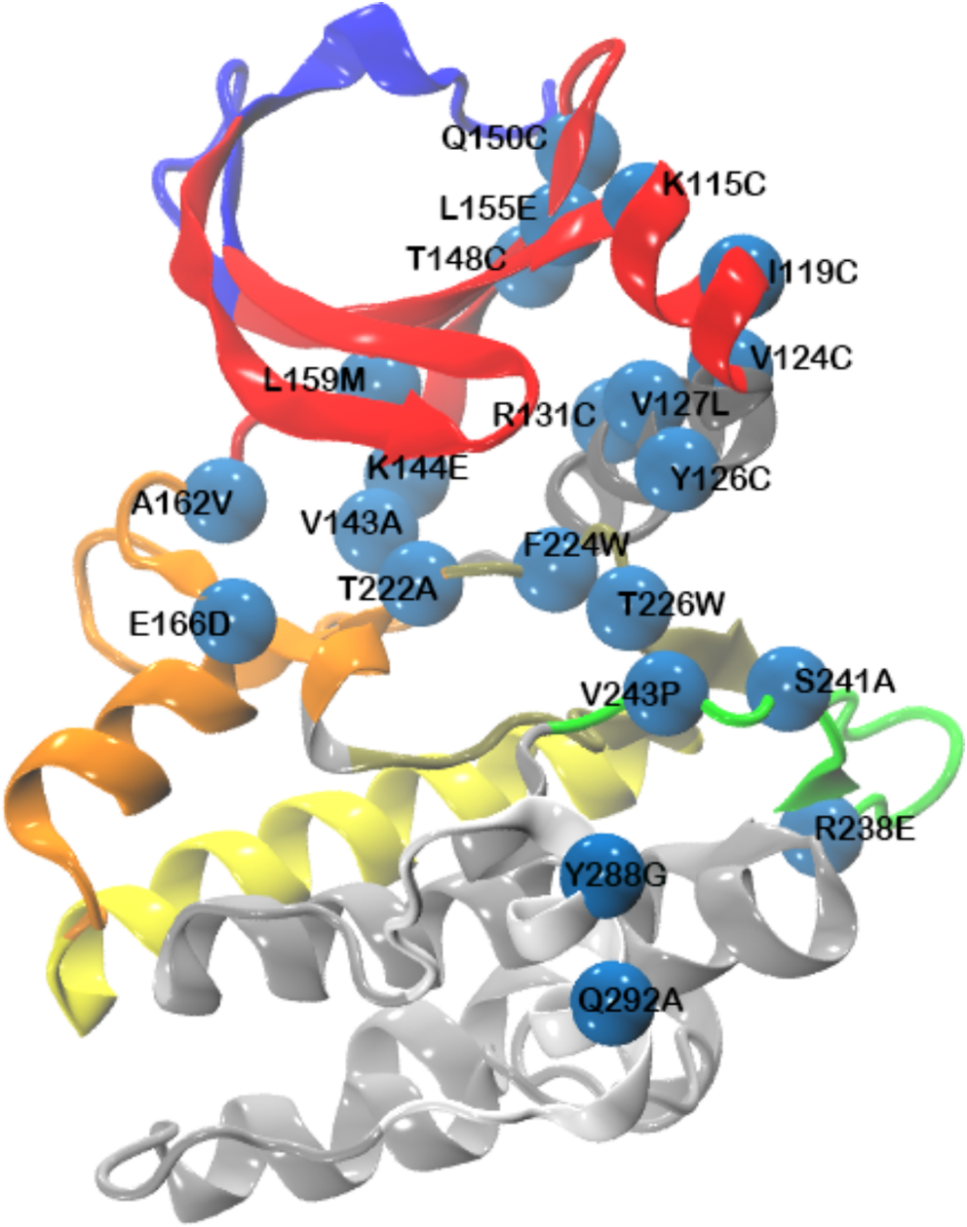
Experimentally reported mutations that affect enzyme activity or its response to allosteric modulators are shown as blue beads. Colors of protein represent communities as shown in Figure 2A.

To validate our computational findings, experimentally reported mutations that impact allosteric activity and modulator binding in PIF-pocket are mapped onto the PDK1 structure, as shown in Figure 6. Mutations are found to spread across various communities, except for the silver and yellow communities. However, the silver community interacts extensively with other communities, and these interactions are highly correlated with the modulator’s allosteric activity profile, indicating that this community is an integral part of the allosteric network. Mutations located far away from the allosteric or the active site (e.g., those in the white community) may exert their effects allosterically through the silver community. Direct experimental mutagenesis in the silver community is needed to further validate its role.

The proximal mutations (Y126C, V127L, V124C, I119C, K115C, Q150C, L155E, T148C)^37,40,45^ to the allosteric site are found in the gray community (Y126C, V127L, V124C) and the red community (I119C, K115C, Q150C, L155E, T148C). Mutations located near the ATP binding active site, such as L159M, A162 V, E166D, K144E, V143A, T222A, F224W, and T226W, are located in three distinct communities: the red community (L159M), the orange community (A162V, E166D, K144E, V143A)unity (T222A, F224W, T226W).^35,61^ These mutations may influence substrate or modulator binding directly, or through a short-range allosteric effect.

Valeri et al. observed that the F224W and T226W mutants exhibited a 3-fold decrease in specific activity compared to the wild-type protein. Biochemical analysis indicated that both the wild-type and F224W proteins were activated by PIFtide and small molecules, whereas the T226W mutant did not respond to activation by either PIFtide or small compounds.^35^ T222 and F224 are found to form a top-three correlated residue–residue contact between the orange and the tan community (Figure 5B), displaying a negative correlation of −0.65. Intriguingly, the overall contact between the orange and tan communities is positively correlated with the allosteric activity of modulators, with a correlation of 0.7 (Figure 4A). This suggests a frustrated relationship between local intercommunity residue-wise interactions and modulator activity.

Mutations Y288G and Q292A, situated over 30 Å from the PIF-pocket at the base of the large lobe, impair the crystallographic packing of the PIF-pocket.^35^ These mutations occur in the white-colored community, whose interaction with the silver community exhibits an overall negative (−0.7) correlation with modulator activity. As discussed above, residue–residue interactions between these two communities likely influence the allosteric site in a collective manner. Mutations V243P, R238E, and S241A regulate the kinase activity of PDK1 upon binding to the 14–3–3 protein.^62^ These mutations are situated within the green community, in proximity to the silver and tan communities, and are not involved in either the ATP-binding pocket or the PIF pocket. Intriguingly, V243, and R238 are predicted to be involved in residue–residue contacts ranked among the top three in residue-wise correlation (Figure 5A). Although S241 is not identified to be in the top-3 contacts, the contact involving S241 exhibiting a high negative correlation value of −0.75, as noted in the Supporting Information (Table S1).

## Conclusions

In this study, we generate conformational ensembles for the wild-type PDK1 kinase in the absence and presence of allosteric modulators bound within the PIF-pocket. By comparing these ensembles, we investigated the influence of small-molecule binding in the PIF pocket on enzyme activity through allosteric regulation. This analysis, employing tools like contact statistics, dCNA-based residue community analysis, and multiensemble contact network analysis, yielded detailed insights into the dynamic conformational changes induced by small-molecule interactions with the enzyme. The advanced analysis of contact-based residue communities integrates multiple conformational ensembles into a single network, effectively linking conformational dynamics with function (activity of enzyme). The results of residue-wise contact correlations were compared with existing experimental mutations, validating the identified function-related residues that could be targeted in future experiments. Our work provides valuable insights into the allosteric mechanisms of these experimentally verified mutations.

## Supporting Information Available

RMSF of modulator-free and modulator-bound complexes (Figure S1); modularity of PDK1 kinase relative to the number of communities (Figure S2); correlation plot between red and pink, gray and pink, tan and green communities’ residue–residue contact relative to log(*K*) values when considering all modulators (Figure S3); correlation plot between orange and red, yellow and green communities’ residue–residue contact relative to log(*K*) values when considering all modulators with correlation <0.1 (Figure S4); correlation plot between red and pink, gray and pink, tan and green communities’ residue–residue contact relative to log(*K*) values when excluding modulators containing experimental *K*_d_ values only (Figure S5) (PDF)
