# Supplementary material for: Uncovering the Role of Distal Regions in PDK1 Allosteric Activation
Source: ACS Bio Med Chem Au. 2025 Mar 24;5(2):299–309. doi: 10.1021/acsbiomedchemau.5c00025 (PMC12006859; doi:10.1021/acsbiomedchemau.5c00025)
Supplement: Supplementary file 1 — bg5c00025_si_001.pdf [file bg5c00025_si_001.pdf]

Supporting Information

Uncovering the Role of Distal Regions in PDK1 Allosteric Activation

Nagaraju Mulpuri[1], Xin-Qiu Yao[2], and Donald Hamelberg[1]*

[1]Department of Chemistry, Georgia State University, Atlanta, Georgia 30302-3965, USA.

[2]Department of Chemistry, University of Nebraska at Omaha, Omaha, Nebraska 68182-0266, USA.

*Correspondence to: Dr. Donald Hamelberg; Department of Chemistry, Georgia State University, P. O. Box 3965, Atlanta, GA 30302-3965, USA.

Telephone: (404) 413-5564. E-Mail: dhamelberg@gsu.edu.

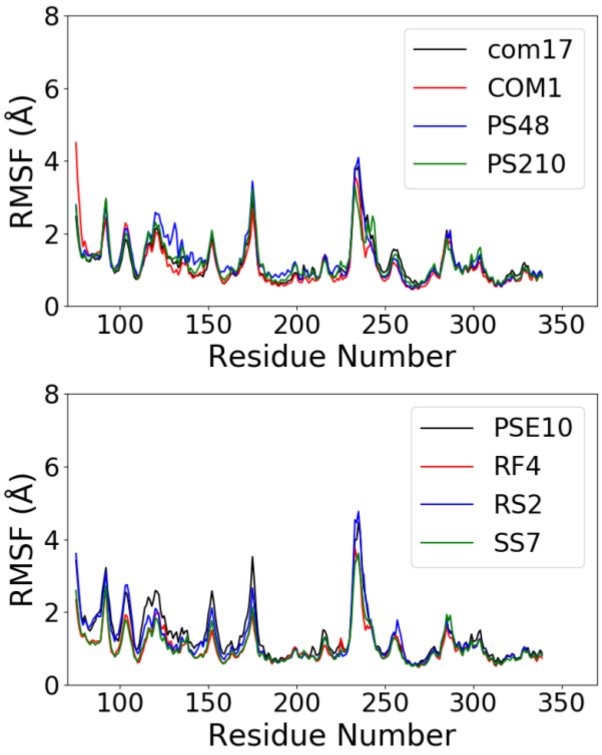

Figure S1: average RMSF of PDK1 complex with modulators, calculated using last 1.5-µs MD simulations.

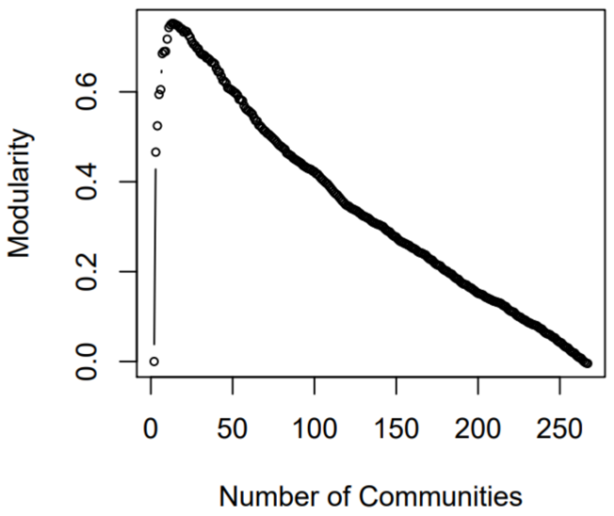

Figure S2: Identifying the optimal number of communities that maximize the network modularity. Modularity were calculated for all modulators considered in multiple distance contact network analysis.

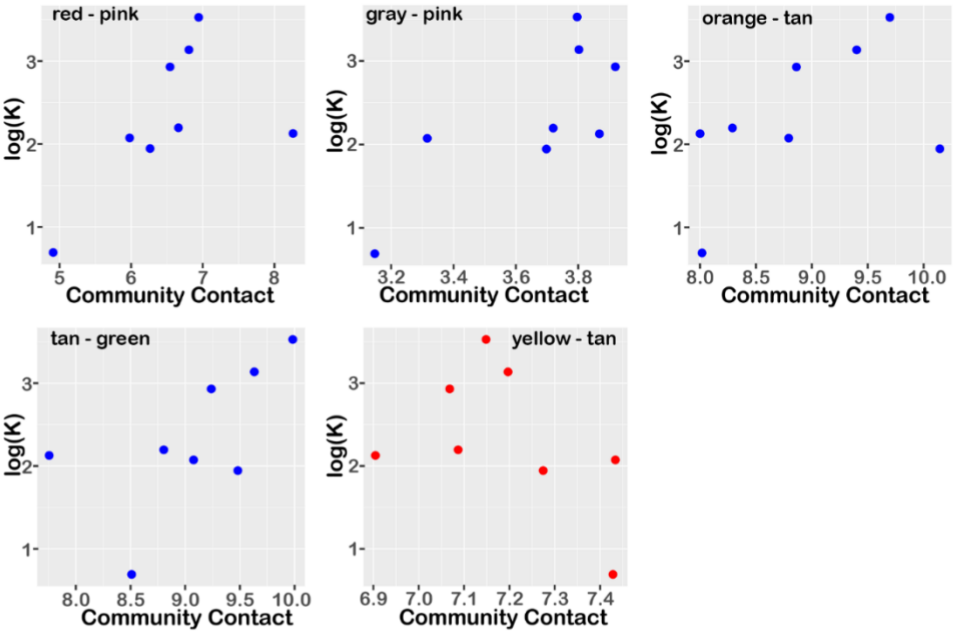

Figure S3: Residue communities and correlation analysis between community-community contacts and all modulator's allosteric activity. Blue color graphs represent positive correlation and red color graphs for negative correlation. Community names are given in black text. This figure is related to Figure 2 in the main text.

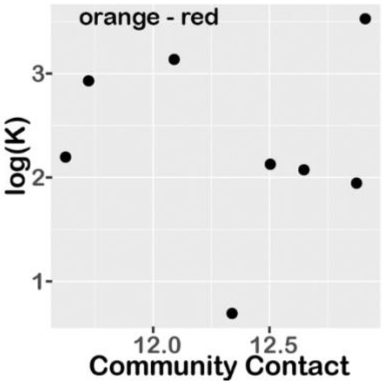

Figure S4: Residue communities and correlation analysis between community-community contacts and all modulator's allosteric activity. Community names are given in black text. This figure is related to Figure 2 in the main text.

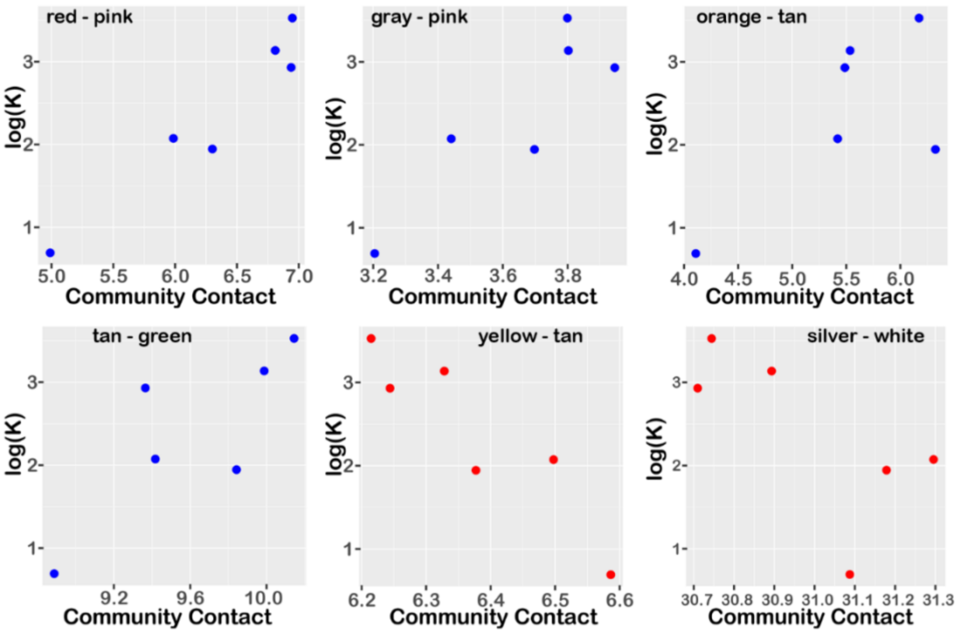

Figure S5: Residue communities and correlation analysis between community-community contacts and modulator's allosteric activity excluding Kd. Blue color graphs represent positive correlation and red color graphs for negative correlation. Community names are given in black text. This figure is related to Figure 3 in the main text.

Table S1: residue-wise correlations calculated using residue-residue contact statistics when considering modulators excluding the ones that contain Kd only. Full list of correlation values in the range >0.5 or < -0.5 are shown here for orange-tan, tan-green, and yellow-tan communities (see Figure 5 in the main text). The residue-wise correlation values of red-pink, gray-pink, and white-silver communities are out of the range of > 0.5 or < -0.5, hence not listed here.

orange and tan community

Res ID	ResID	Correlation
207	211	0.723511
205	209	0.688133
206	210	0.680554
203	210	0.66401
207	210	0.617718
206	211	0.581903
209	223	0.574502
205	210	0.544421
203	211	0.528023
222	224	-0.61618
204	210	-0.62969

tan and green community

ResID	ResID	Correlation
204	244	0.84672
202	238	0.824264
230	232	0.739473
204	243	0.712273
202	239	0.701456
200	231	0.640767
230	236	0.547925
203	241	0.542616
226	244	0.540911
230	233	0.509981
202	237	-0.52097
205	241	-0.75233
226	240	-0.75907
205	240	-0.7722
203	240	-0.78939
207	240	-0.8034
206	242	-0.8034
200	234	-0.83991
207	243	-0.84128
204	240	-0.90909
207	242	-0.98622

yellow and tan community

ResID	ResID	ResID
199	201	0.799763
199	229	0.54198
193	203	-0.61979
197	202	-0.62479
197	203	-0.68854
196	227	-0.74545
195	224	-0.8034
189	207	-0.8034
198	230	-0.8034
197	230	-0.94558
